# The Oncogenic Activity of *miR-29b-1-5p* Induces the Epithelial-Mesenchymal Transition in Oral Squamous Cell Carcinoma

**DOI:** 10.3390/jcm8020273

**Published:** 2019-02-24

**Authors:** Miyako Kurihara-Shimomura, Tomonori Sasahira, Hiroyuki Shimomura, Chie Nakashima, Tadaaki Kirita

**Affiliations:** 1 Department of Molecular Pathology, Nara Medical University, Kashihara, Nara 634-8521, Japan; miyako@naramed-u.ac.jp (M.K.-S.); c-nakashima@narmaed-u.ac.jp (C.N.); 2 Department of Oral and Maxillofacial Surgery, Nara Medical University, Kashihara, Nara 634-8521, Japan; hiroz@naramed-u.ac.jp (H.S.); tkirita@naramed-u.ac.jp (T.K.)

**Keywords:** miR-29b-1-5p, oral cancer, epithelial-mesenchymal transition, prognosis

## Abstract

Background: The relationship between *miR-29b-1-5p* and c-Met proto-oncogene in oral squamous cell carcinoma (OSCC) remains to be investigated. This study aimed to reveal the role of *miR-29b-1-5p* in the pathogenesis of OSCC using molecular and biological analyses. Methods: We investigated the expression of *miR-29b-1-5p*, c-Met, and markers of the epithelial-mesenchymal transition (EMT) in the tissues of 49 patients with OSCC and in human OSCC cells with different tumorigenicity. Further, we determined the effects of *miR-29b-1-5p* on the phenotypes of OSCC cell lines. Results: The expression levels of *miR-29b-1-5p* in most patients with OSCC were higher than those of the normal oral epithelium. In OSCC, upregulation of *miR-29b-1-5p* significantly correlated with histological grade, the EMT, and the immunohistochemical grade, indicated by c-Met expression. The prognosis was poor for patients with *miR-29b-1-5p* expression and coexpression of *miR-29b-1-5p* and c-Met. In OSCC cells exhibiting the EMT phenotype, knockdown of miR-29b-1-5p suppressed the EMT, which was recovered by enforced expression of c-Met. Further, the mRNA encoding cadherin 1 (*CDH1*) was a direct target of *miR-29b-1-5p*. Conclusions: Our results suggest that *miR-29b-1-5p* acts as an oncogenic miRNA that synergizes with c-Met to induce the EMT of OSCC cells.

## 1. Introduction

Cancer of the head and neck, including oral squamous cell carcinoma (OSCC), is the sixth most common malignant tumor worldwide, with approximately 529,500 patients and 292,300 deaths annually, which represent approximately 3.8% of all cancer cases and 3.6% of cancer-related deaths [1,2]. For example, the yearly toll in the United States is approximately 51,540 new diagnoses and 10,030 OSCC-related deaths [3]. OSCC is highly malignant because of its ability to invade surrounding tissues and metastasize, such that the overall five-year survival rate is <50%, which has not increased during the past 30 years [4]. Although >80% of patients with early-stage OSCC are cured, approximately 70% of patients with advanced OSCC do not survive [4]. Efforts to address this dismal outcome must involve identifying the molecular mechanism of the pathogenesis of OSCC to achieve early detection and improve treatment strategies.

MicroRNAs (miRNAs) (approximately 18–25 nucleotides) are noncoding single-stranded RNAs that inhibit gene expression by binding to the 3′-untranslated regions (UTR) of target mRNAs [5]. MiRNAs are transcribed by RNA polymerase II from DNA into primary miRNA (pri-miRNA) molecules, and the primary transcription product, designated long pri-miRNA, is processed by drosha ribonuclease III (DROSHA) and DiGeorge Syndrome Critical Region 8 (DGCR8) to generate precursor miRNA (pre-miRNA). Exportin 5 (XPO5) mediates the export of a pre-miRNA from the nucleus to the cytoplasm where dicer 1, ribonuclease III (DICER1) converts it into a short double-stranded miRNA. A single strand of mature miRNA is incorporated along with argonaute RISC catalytic component 2 (AGO2) into an RNA-induced silencing complex (RISC) that inhibits the expression of a target mRNA [5,6].

The *miR-29b-1-5p* molecule represents the 5′-miRNA generated from the same stem-loop as the 3′ miRNA, designated *miR-29b-1*, and the sequence encoding *miR-29* resides within 7q32.3 locus of the human genome [7,8]. The mechanism of transcriptional regulation of *miR-29b-1-5p* is identical to that of *miR-29b-1*, although they differ with respect to their processing and maturation [8]. Evidence indicates that *miR-29b-1* acts as a tumor suppressor in many malignancies [9]. However, *miR-29b-1-5p* may mediate other processes in cancer cells. For example, expression of *miR-29b-1-5p* is significantly diminished in sunitinib-resistant renal carcinoma cells (RCCs) [10]. Further, *miR-29b-1-5p* is downregulated in basal-like and triple-negative breast cancers [8,11]. Conversely, higher levels of expression of *miR-29b-1-5p* are associated with the proliferation of bladder cancer cells [12], and overexpression of *miR-29b-1-5p* contributes to the development of gastric cancers from premalignant adenomas [13]. The full spectrum of *miR-29b-1-5p* functions in malignancies remains to be determined.

The gene encoding the c-Met is located on human chromosome 7q31.2 and mediates the progression of OSCC [14,15,16], and c-Met is associated with the induction of the epithelial-mesenchymal transition (EMT) in certain malignancies [17,18,19]. Cancer cells that undergo the EMT lack epithelial cell-to-cell contacts, which are associated with the suppression of CDH1 expression and increased expression of mesenchymal markers such as VIM [17]. Further, upregulation of the expression of the transcription factor SNAI1 is required for the maintenance of the EMT of cancer cells [19].

However, there are no studies, to our knowledge, which investigate the relationship between *miR-29b-1-5p* and c-Met in cancer cells. To address this deficiency in our knowledge, we hypothesized that coordinate regulation of *miR-29b-1-5p* and c-Met meditates the EMT of OSCC cells. To provide support for this theory, here we evaluated expression and functional roles of *miR-29b-1-5p* in OSCC.

## 2. Materials and Methods

### 2.1. Surgical Specimens

Formalin-fixed, paraffin-embedded (FFPE) specimens were acquired from 49 patients with OSCC (20 men and 29 women; mean age: 66 (46–91) years) for retrospective expression analysis. Controls included five samples of normal oral mucosal tissues, including the epithelium, which were acquired from three men and two women (mean age: 42.5 (36–45) years). The tissues were randomly selected from patients treated at Nara Medical University Hospital, Kashihara, Japan. Preoperative treatment was not administered to all patients. Written informed consent was obtained from individual patients for the use of their tissue samples. Tumor stages of patients with OSCC were classified according to the criteria of the Union for International Cancer Control TNM classification system (8th edition). Further, the histological grades of the OSCCs were classified according to the criteria of the World Health Organization.

Medical records and prognostic follow-up data were acquired from the hospital’s database. The follow-up period was 248–1894 days (mean, 1126 days; median, 998 days). To evaluate the association between *miR-29b-1-5p* expression and patients’ clinicopathological characteristics, patients were allocated into two groups according to their expression levels of *miR-29b-1-5p* as follows: Values higher or lower than the mean value of the entire group [4]. Moreover, specimens with decreased expression of *CDH1* and increased expression of *VIM* were classified as tissues undergoing the EMT [20]. The Medical Ethical Committee of Nara Medical University approved this study (approval number: 719). The study protocol for the use of human samples was in accordance with the provisions of the Declaration of Helsinki.

### 2.2. Laser Capture Microdissection

Laser capture microdissection (LCM) was performed to specifically select OSCC cells for the preparation of small RNAs. Tissue sections (7 μm) were prepared from each paraffin block and stained using hematoxylin and eosin. A PixCell II laser capture microdissection microscope (Arcturus, Mountain View, CA, USA) was used to capture and transfer cells for microdissection according to the manufacturer’s instructions. Approximately 5000 tumor cells were acquired from each tissue sample. Small RNAs were extracted from FFPE specimens using an miRNeasy FFPE Kit (Qiagen, Venlo, Netherlands) [4].

### 2.3. Immunohistochemistry

Consecutive 3-μm sections were cut from each block, and an EnVision+ Dual Link System (Dako, Carpinteria, CA, USA) was used to perform immunohistochemical analyses. An immunoperoxidase technique was performed after antigen retrieval employing microwave treatment (95 °C) in citrate buffer (pH 6.0) for 45 min. After endogenous peroxidase activity was inhibited using a solution of 3% H_2_O_2_ in methanol, specimens were incubated in a solution of 10% skim milk (Morinaga Milk, Tokyo, Japan) for 20 min to prevent nonspecific antibody reactions. Antibodies against c-Met (Invitrogen, Carlsbad, CA, USA), CDH1 (BD Biosciences, San Jose, CA, USA), and vimentin (VIM) (Dako) were diluted to 0.5 μg/mL. After incubation for 2 h at room temperature, specimens were treated for 30 min at room temperature with the secondary antibody. The specimens were treated with a solution of diaminobenzidine (DAB) (Dako) and counterstained with Meyer’s-hematoxylin (Sakura Finetek, Tokyo, Japan). The immunoreactivities of c-Met in the samples were classified according to Allred’s score (AS) [21] as follows: grade 0, AS = 0; 1 AS = 2–4; and 2 for AS = 5–8. Grade-2 samples were classified as c-Met overexpression [22].

### 2.4. Cell Culture

Human OSCC cell lines KON, HSC3, and HSC4 were acquired from the Health Science Research Resources Bank and cultured in Dulbecco’s Modified Eagle’s Medium (DMEM) (Wako Pure Chemical, Osaka, Japan) containing 10% fetal bovine serum (Nichirei Biosciences, Tokyo, Japan) in an atmosphere containing 5% CO_2_ at 37 °C. KON and HSC3 cells have high metastatic potential, HSC4 cells have low metastatic potential, and HSC2 cells are unable to metastasize or invade [22].

### 2.5. Transient Transfection

We purchased mirVana *miR-29b-1-5p* mimics, mirVana *miR-29b-1-5p* inhibitor, pre-miR negative control#1, and anti-miR negative control#1 from Ambion (Austin, TX, USA). Overall, 10 nM of siRNA, 10 nM of pre-miR, and 20 nM of anti-miR were used to transfect cells in the presence of Lipofectamine 3000 (Invitrogen) according to the manufacturer’s recommendations. An anti-c-Met antibody (2 μL/mL) (Invitrogen) was employed to neutralize human recombinant MET (10 μM, rhMET) (Abcam, Cambridge, Cambridgeshire, UK) added to the culture medium.

### 2.6. Quantitative Reverse-Transcription Polymerase Chain Reaction (qRT-PCR) and qPCR

Total and small RNAs were extracted using TRIzol (Invitrogen) or the mirVana miRNA Isolation Kit (Ambion); and 1 and 10 ng of total and small RNAs, respectively, were synthesized using a ReverTra Ace qRT Kit (Toyobo, Osaka, Japan) or a TaqMan MicroRNA Reverse Transcription Kit (Applied Biosystems, Foster City, CA, USA), respectively. Genomic DNA was extracted using TRIzol (Invitrogen) reagent.qPCR and qRT-PCR analyses were performed using a StepOne Plus Real-Time PCR Systems (Applied Biosystems) combined with a TaqMan Fast Universal PCR Master Mix (Applied Biosystems), and the data were analyzed using a relative standard curve-quantification method. PCR conditions followed the manufacturer’s protocols. Glyceraldehyde-3-phosphate dehydrogenase (*GAPDH*) mRNA and eukaryotic RNA, 18S ribosomal 1 (*RNA18S1*) were amplified to serve as internal controls. TaqMan Gene Expression Assays were used to detect the mRNAs as follows: *c-Met*, *CDH1*, *VIM*, snail family transcriptional repressor 1 (*SNAI1*), *GAPDH*, and *RNA18S1*. TaqMan MicroRNA Assays kits to detect hsa-*miR-29b-1-5p* were purchased from Applied Biosystems. All PCR assays were performed in triplicate.

### 2.7. Immunoblotting

Whole-cell lysates were prepared using the M-PER Mammalian Protein Extraction Reagent (Thermo Fisher Scientific, Rockford, IL, USA). Lysates (50 µg) were subjected to western blotting using 12.5% SDS-PAGE and then electrophoretically transferred to polyvinylidene fluoride membranes (Thermo Fisher Scientific). The membranes were incubated with antibodies against c-Met (Invitrogen), CDH1 (BD Biosciences), VIM (Dako), and SNAI1 (R&D Systems, Minneapolis, MN, USA) and then with a peroxidase-conjugated IgG (Medical & Biological Laboratories, Nagoya, Japan). Immune complexes were visualized using an ECL Western Blotting Detection Kit (GE Healthcare, Amersham Place, UK). An anti-GAPDH antibody (Santa Cruz Biotechnology, Inc., Dallas, TX, USA) served as an internal control.

### 2.8. Luciferase Reporter Assay

The sequences of the wild-type (wt) and mutant (mut) *CDH1* 3′-untranslated regions (UTRs) comprising *miR-29b-1-5p* binding sites were synthesized and inserted into the psiCHECK vector (Promega Corporation, Madison, WI, USA) that expresses firefly luciferase. Cells were transfected with this reporter plasmid and an *miR-29b-1-5p* mimic/inhibitor, or a control miRNA in the presence of Lipofectamine 3000 (Invitrogen). Experiments were performed in triplicate. Luciferase activities were determined using the Dual-Luciferase Reporter System (Promega Corporation) according to the manufacturer’s instructions.

### 2.9. Statistical Analysis

Statistical analysis was performed using JMP13 software (SAS Institute, Cary, NC, USA). The significance of differences was evaluated using Fisher’s exact test, the χ^2^ test, the Mann-Whitney *U*-test, and the Student *t*-test. Disease-free survival (DFS) and overall survival (OS) were analyzed using the Kaplan-Meier method, and differences between groups were calculated using a log-rank test. *p* < 0.05 indicates a significant difference.

## 3. Results

### 3.1. Expression of miR-29b-1-5p and c-Met and EMT Status of Patients with OSCC

**Figure 1:**
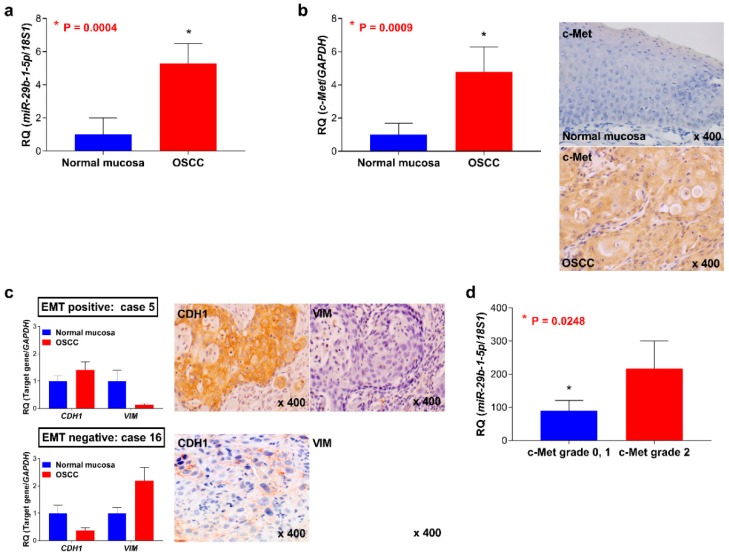
Analyses of the expression of *miR-29b-1-5p* and c-Met and the status of the epithelial- mesenchymal transition (EMT) in patients with oral squamous cell carcinoma (OSCC). (**a**) Levels of expression of *miR-29b-1-5p* in OSCC and normal oral mucosal tissues. Representative examples of c-Met expression (**b**) and EMT status (**c**) in OSCC tissues. (**d**) The levels of *miR-29b-1-5p* were significantly associated with the c-Met immunohistochemical grade (*p* = 0.0248).

The expression levels of *miR-29b-1-5p* and *c-Met* were higher in most samples of OSCC than those of normal oral epithelial tissues (*p* = 0.0004) (Figure 1a,b). Representative data for c-Met expression and EMT markers show that 55.1% (27/49) and 38.8% (19/49) of OSCC samples were positive for c-Met overexpression and EMT markers, respectively (Figure 1b,c).

### 3.2. Association between the Expression of miR-29b-1-5p and Clinicopathological Characteristics of Tissue Samples of Patients with OSCC

The associations between *miR-29b-1-5p* or c-Met expression and the clinicopathological characteristics of patients with OSCC are summarized in Table 1. Increased expression of *miR-29b-1-5p* was significantly associated with the degree of histological differentiation of OSCC cells (*p* = 0.0031). Upregulation of *miR-29b-1-5p* was detected in 18 of 19 (94.7%) samples of patients with OSCC that expressed the EMT phenotype. Conversely, 12 of 30 (43.3%) samples of patients with OSCC that did not exhibit the EMT phenotype had decreased levels of *miR-29b-1-5p* (*p* = 0.0002). c-Met was detected in 14 of 19 (73.7%) patients that exhibited the EMT phenotype, with lower rates among patients (13 of 30 (43.3%)) that did not exhibit the EMT phenotype (*p* = 0.0453). The levels of *miR-29b-1-5p* significantly correlated with the immunohistochemical grade of c-Met (*p* = 0.0248) (Figure 1d). There were no significant correlations between *miR-29b-1-5p* or c-Met expression with other clinicopathological factors of the patients with OSCC.

### 3.3. Prognosis of Patients with OSCC

**Figure 2:**
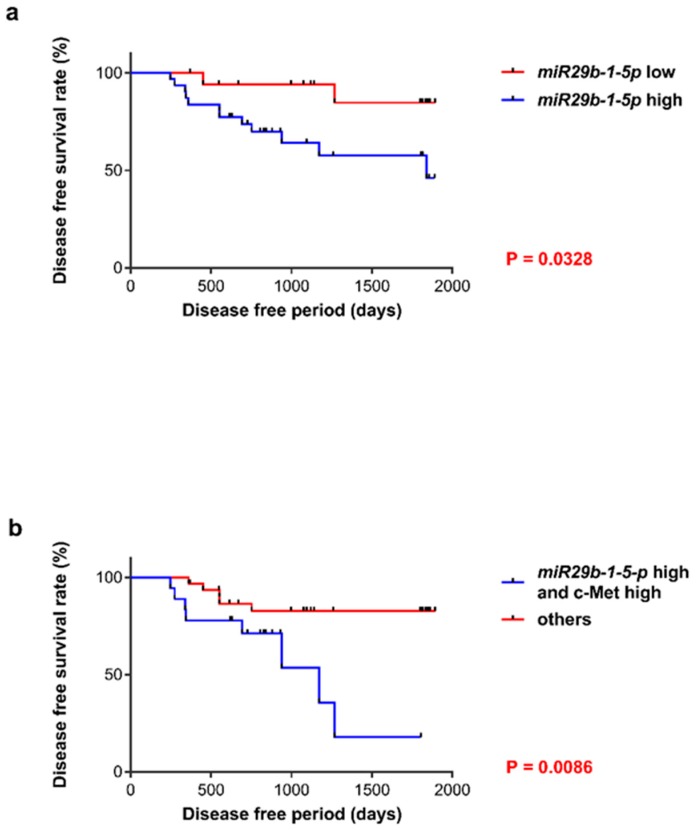
Patients with OSCC. (**a**) Patients with elevated levels of *miR-29b-1-5p* experienced significantly shorter DFS than those with relatively lower levels of miR-29b-1-5p expression (*p* = 0.0328). (**b**) Patients with OSCC along with the concurrent expression of *miR-29b-1-5p* and c-Met experienced significantly shorter DFS than other patients included in this study (*p* = 0.0086).

During follow-up, 21 of the 49 (42.9%) patients with OSCC presented with a local or metastatic recurrence of their tumors, and 13 of the 49 (26.5%) patients experienced OSCC-specific mortality. The DFS of patients with OSCC revealed that a poor outcome was not significantly associated with the levels of expression of MET. However, patients that expressed *miR-29b-1-5p* (*p* = 0.0328) concomitantly with high levels of *miR-29b-1-5p* and MET experienced a significantly higher frequency of unfavorable outcomes (*p* = 0.0086) than those of other patients (Figure 2a,b). 

### 3.4. Expression of miR-29b-1-5p and c-Met and EMT Status in OSCC cells

**Figure 3:**
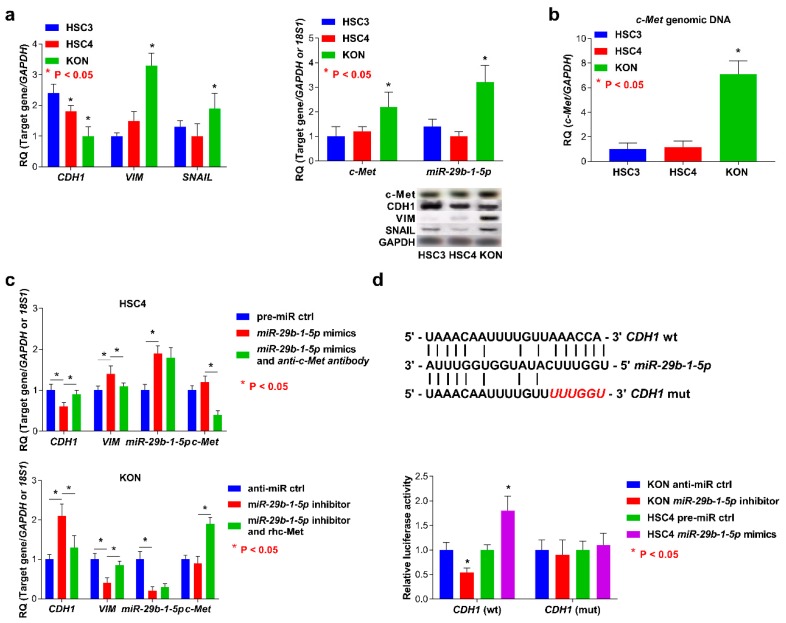
Analysis of the status of the EMT and c-Met and *miR-29b-1-5p* expression and function of *miR-29b-1-5p* in OSCC cells. (**a**) The levels of expression of *miR-29b-1-5p* and *c-Met* in KON cells were higher than those of HSC3 and HSC4 cells (left). Upregulation or downregulation of *CDH1* or *SNAI1* and *VIM* was detected in KON cells, respectively (right). (**b**) *MET* genomic DNA was amplified compared with the copy numbers measured in HSC3 and HSC4 cells. (**c**) Induction of the EMT was mediated through the interaction of *miR-29b-1-5p* with c-Met in OSCC cells. (**d**) The 3′-UTR of *CDH1* contains *miR-29b-1-5p* binding sites. Reporter constructs showing wild-type (wt) and mutated (mut) *CDH1* 3′ UTR sequences (top). Luciferase activity of OSCC cells transfected with reporter vectors containing the wt or mut 3′-UTR of *CDH1*. Error bar: standard deviation (bottom). RQ: relative quantitation.

Higher levels of *miR-29b-1-5p* and *MET* were detected in KON cells than those of HSC3 and HSC4 cells (Figure 3a). In KON cells, the expression levels of *CDH1* were increased, and the levels of *SNAI1* and *VIM* were lower than those detected in HSC3 and HSC4 cells (Figure 3a). Further, amplification of *MET* genomic DNA was detected at higher levels in KON cells than that in HSC3 and HSC4 cells (Figure 3b). We also verified that *miR-29b-1-5p* regulates morphological change and *CDH1* expression in OSCC cells. Treatment with *miR-29b-1-5p* mimics induced the EMT in HSC4 cells, which was demonstrated through the detection of elevated levels of *miR-29b-1-5p* and *VIM* and downregulation of *CDH1*. Conversely, lower levels of *miR-29b-1-5p* and *VIM* and higher levels of *CDH1* were detected in KON cells treated with the *miR-29b-1-5p* inhibitor (Figure 3c). The expression of the molecules analyzed above and the EMT phenotypes were inhibited through combined treatment of HSC4 or KON cells with *miR-29b-1-5p* mimics and rhc-Met, or the *miR-29b-1-5p* inhibitor and an anti-c-Met antibody, respectively (Figure 3c). Surprisingly, *CDH1* was also identified as potentially involved in the target gene of *miR-29b-1-5p* by online programs mivroRNA.org (http://www.microrna.org/) (Figure 3e). In the OSCC cells with wild-type *CDH1* 3′-UTR, luciferase activity was altered in the presence of an *miR-29b-1-5p* mimic or an inhibitor compared with control cells. This effect was completely inhibited when the *miR-29b-1-5p* binding site was deleted from the *CDH1* 3′-UTR (Figure 3e). There was no detectable association between altered expression of *MET* and the alterations in the expression of *miR-29b-1-5p*.

## 4. Discussion

Here we show that the levels of expression of *miR-29b-1-5p* and c-Met were significantly associated with the phenotypes of specimens harvested from the tissues of patients with OSCC. Specifically, patients who tested positive for miR-29b-1-5p and concurrent expression of *miR-29b-1-5p* and c-Met had unfavorable prognoses compared with those of other patients included in the present study. We analyzed OSCC cell lines and found that the induction of the EMT was regulated by synergic or cooperative effects of *miR-29b-1-5p* and c-Met. Further, we found that *CDH1* was a direct target of *miR-29b-1-5p*.

The small sizes of noncoding RNAs and miRNAs contribute to their stabilities in FFPE specimens [4,23]. Most published analyses of miRNA expression in clinical specimens employed frozen tissues containing epithelial cells, stromal cells, and cancer cells. For example, *miR-29b-1-5p* expression is increased during the proliferation of myoblasts [24], and therefore, the use of fresh frozen samples that include numerous cell types, such as muscle cells, may lead to confusion, likely causing the investigators to misinterpret their data. Hence, we used LCM to specifically select cancer cells from FFPE specimens for qRT-PCR analysis of the expression of *miR-29b-1-5p*.

*MiR-29b-1* performs multiple functions in malignancies, and overexpression of *miR-29b-1* is associated with clinical stage, nodal metastasis, poor prognosis, proliferation, and downregulation of C-X3-C motif chemokine ligand 1 (*CX3CL1*), which is directly targeted by miR-29b-1 in OSCC cells [25]. Conversely, few published studies, to our knowledge, address the potential associations between *miR-29b-1-5p* and the phenotypes of cancer cells. Although decreased expression of *miR-29b-1-5p* occurs in breast cancer and RCCs [8,10,11], higher levels of expression of *miR-29b-1-5p* influence cell proliferation and the adenoma–carcinoma transition in bladder cancer and gastric carcinoma, respectively [12,13].

miRNAs can act as an oncomiR or as a tumor suppressor through their interactions with target mRNAs that influence the interactions between cancer cells and the host’s immune system as well as interactions with stromal cells, oncoviruses, and mRNAs that encode the components of signaling pathways that mediate resistance to therapeutic agents [26]. Further, miRNAs regulate gene expression by binding to the 3′-UTR of target mRNAs. Therefore, *miR-29b-1-5p* may activate multiple target genes.

The expression of *CDH1* was downregulated by *miR-29b-1-5p* in this study. Conversely, other *miR-29b-1-5p*-target genes are not well documented. The function of *miR-29b-1-5p* in cancer cells is controversial; therefore, further studies are required to better understand the role of *miR-29b-1-5p* and its target genes in the pathogenesis of OSCC.

The *c-Met*, which maps to human chromosome 7q31.2, acts as oncogene in OSCC [14,15,16]. In patients with gastric carcinoma, *MET* is frequently amplified and is associated with the development and progression of these tumors [27,28]. Nonetheless, loss of heterozygosity (LOH) occurs at the c-Met locus in stomach cancer [29]; therefore, their claims are contradictory. Here, our analyses revealed amplification of *MET* and upregulation of c-Met mRNA in OSCC cells. Further, LOH at 7q31.2 was detected in OSCC cells. The expression of c-Met is also associated with the EMT of patients with OSCC and therefore may be beneficial as a tumor marker of this disease.

*MiR-9*, *miR-21*, the *miR-31* family, *miR-155-5p, miR-196a*, and *miR-372* activate the EMT in OSCC [30]. However, there are no published studies, to our knowledge, of the role of *miR-29b-1-5p* in regulating the EMT. We show here that the expression levels of *miR-29b-1-5p* and c-Met were significantly associated with the EMT status of patients with OSCC. Further, inhibition of *miR-29b-1-5p* expression diminished the ability of KON cells, which have acquired EMT phenotype, to undergo the EMT. Further, upregulation of *miR-29b-1-5p* led HSC4 cells to undergo the EMT. These changes were restored by treatment of KON cells or HSC4 cells with rhMET or an anti-c-Met antibody, respectively.

Our results indicate that direct or indirect interactions between *miR-29b-1-5p* and c-Met are required for OSCC cells to undergo the EMT. However, *miR-29b-1* downregulates the expression of the gene encoding DNA methyltransferase 3 beta (*DNMT3B*) and inhibits the EMT and the invasiveness of OSCC cell lines [30,31]. Further studies are therefore required to better understand the relationships between the activities of members of the *miR-29* family and the EMT of cancer cells.

## 5. Conclusions

In conclusion, we prevent compelling evidence that supports the conclusion that *miR-29b-1-5p* acts as an oncomiR and that *miR-29b-1-5p* and MET cooperate to induce OSCC cells to undergo the EMT. Moreover, *miR-29b-1-5p* negatively regulated *CDH1* expression.

*MiR-29b-1-5p* may be detectable in serum, saliva and other samples and, therefore, may serve as a therapeutic target as well as a marker for patients with OSCC. Molecularly targeted therapies are available for treating patients with OSCC, except for cetuximab, a chimeric monoclonal antibody against the epidermal growth factor receptor (EGFR), and nivolumab, an antibody that inhibits activity of the programmed cell death 1 (PD-1) receptor [32,33,34]. Therefore, molecular markers that are practical for use in the diagnosis and treatment of OSCC are urgently required. Although further preclinical and large clinical studies are required to identify the mechanism of the functions of *miR-29b-1-5p*, our findings indicate that *miR-29b-1-5p* is a key oncomiR and therefore a target for molecular diagnosis and treatment of patients with OSCC.

## Figures and Tables

**Table 1:** Relationship between *miR-29b-1-5p* or c-Met expression and clinicopathological parameters.

Parameters	*miR-29b-1-5p*		c-Met	
	Low	High	Negative	Positive
Gender				
Male	12	10	10	10
Female	10	19	12	17
*p* value	0.7679		0.5741	
Age				
<65	8	10	7	11
>65	10	21	15	16
*p* value	0.5401		0.5647	
Site				
Tongue	9	21	16	14
Other	9	10	6	13
*p* value	0.2416		0.1551	
Alcohol intake				
Habitual drinking	5	15	4	7
Social drinking	5	7	10	10
No drinking	8	9	8	10
*p* value	0.3516		0.7650	
Smoking habit				
Yes	6	8	9	8
No	12	23	13	19
*p* value	0.7442		0.5480	
Histological				
Differentiation *				
Well	15	12	13	14
Mod, Por	3	19	9	13
*p* value	0.0031		0.7738	
T classification				
T1–T2	15	22	18	19
T3–T4	3	9	4	8
*p* value	0.4942		0.5072	
Clinical stage				
I, II	13	16	14	15
III, IV	5	15	8	12
*p* value	0.2298		0.7707	
Nodal metastasis				
Negative	15	19	16	18
Positive	3	12	6	9
*p* value	0.1975		0.7597	
EMT status				
Negative	17	13	17	13
Positive	1	18	5	14
*p* value	0.0002		0.0453	
c-Met expression				
Negative	9	13		
Positive	9	18		
*p* value	0.7665			

Relationship between expression of *miR-29b-1-5p* or c-Met and clinicopathological parameters were calculated by Fisher’s exact test or the chi-square test. T classification and clinical stage were classified according to the TNM classification. * Histological differentiation: Well, well-differentiated squamous cell carcinoma; Mod, moderately differentiated squamous cell carcinoma; Por, poorly differentiated squamous cell carcinoma.
